# K-Ras Mediated Murine Epidermal Tumorigenesis Is Dependent upon and Associated with Elevated Rac1 Activity

**DOI:** 10.1371/journal.pone.0017143

**Published:** 2011-02-15

**Authors:** Michael S. Samuel, Filipe C. Lourenço, Michael F. Olson

**Affiliations:** The Beatson Institute for Cancer Research, Glasgow, United Kingdom; University of Oldenburg, Germany

## Abstract

A common goal for potential cancer therapies is the identification of differences in protein expression or activity that would allow for the selective targeting of tumor vs. normal cells. The Ras proto-oncogene family (K-Ras, H-Ras and N-Ras) are amongst the most frequently mutated genes in human cancers. As a result, there has been substantial effort dedicated to determining which pathways are activated by Ras signaling and, more importantly, which of these contribute to cancer. Although the most widely studied Ras-regulated signaling pathway is the Raf/mitogen-activated protein kinase cascade, previous research in model systems has revealed that the Rac1 GTP-binding protein is also required for Ras-induced biological responses. However, what have been lacking are rigorous *in vivo* Rac1 target validation data and a clear demonstration that in Ras-driven hyperplastic lesions, Rac1 activity is increased. Using a combination of genetically-modified mouse models that allow for the tissue-selective activation or deletion of signaling molecules and an activation-state sensitive Rac1 antibody that detects GTP-bound Rac1, we found that Rac1 contributes to K-Ras induced epidermal papilloma initiation and growth and that Rac1 activity is elevated by oncogenic K-Ras *in vivo*. Previously, it was not practical to assess Rac1 activation status in the most commonly used format for clinical tumor specimens, formalin-fixed paraffin embedded (FFPE) tissues samples. However, this study clearly demonstrates that Rac1 is essential for K-Ras driven epithelial cell hyperproliferation and that Rac1 activity is elevated in tissues expressing mutant oncogenic K-Ras, while also characterizing the activation-state specific Rac1-GTP antibody as a probe to examine Rac1 activation status in FFPE samples. Our findings will facilitate further research on the status of Rac1 activity in human tumors and will help to define the tumor types of the patient population that could potentially benefit from therapies targeting Rac activation or downstream effector signaling pathways.

## Introduction

Members of the Rho family of GTP-binding proteins act as molecular switches, cycling between inactive GDP-bound and active GTP-bound states [1]. The conversion between these states is regulated by guanine nucleotide exchange factors (GEFs) that promote the exchange of GDP for GTP to increase Rho activation [2], and GTPase-accelerating proteins (GAPs) that increase the rate of Rho-catalyzed hydrolysis of the bond between β and γ PO_4_ groups in GTP by providing a catalytic “arginine-finger” resulting in Rho inactivation [3], [4]. In their GTP-bound active state, information is conveyed downstream of Rho family proteins through interactions with effector proteins whose intrinsic activity, subcellular localization and/or association in multimeric protein complexes are altered so as to affect signaling flux [5], [6]. Although best known as central regulators of the actin cytoskeleton and cell morphology [7], Rho GTPases influence a myriad of additional biological processes such as cell proliferation *via* actin-dependent and actin-independent mechanisms [8]. In contrast to the relatively frequent incidence of activating mutations in Ras genes (K-Ras, H-Ras and N-Ras [9]), there are no published reports of activating Rho mutations in human tumors. However, numerous lines of research indicate that Rho proteins do indeed contribute to cancer [5], [6], [10].

Amongst the Rho family, the most thoroughly-characterized are RhoA, Rac1 and Cdc42. To some extent this reflects historical factors and yet in many cases these specific proteins do appear to be vitally important for a number of functions. Early studies using *in vitro* cell culture model systems revealed a contribution of Rac1 to cell cycle regulation [11] and in Ras-mediated transformation [12], [13]. However, given that the reagents used to inhibit endogenous protein signaling were dominant-negative versions of Rac1 that are encumbered by concerns about their specificity, the conclusions of these studies may not be adequately robust [14]. Finally, evidence for a dependence of Ras on Rac1 for biological functions accompanied by evidence of Ras-induced Rac1 activation *in vivo* has remained elusive.

Genetically-modified mouse models that allow for the conditional and tissue-selective deletion or activation of specific signaling proteins are now readily available [15]. In this study, we made use of a strain of mice containing a mutant K-Ras allele (K-Ras^G12D^) that can be conditionally activated by Cre-recombinase mediated excision of a floxed transcription termination (LSL) cassette [16]. It had previously been shown that K-Ras driven lung tumors were dependent upon Rac1. However, whether Rac1 was actually activated by K-Ras or merely necessary at a basal level of activity for K-Ras induced effects was not determined [17]. By crossing *LSL-K-Ras^G12D^* mice with those expressing a Cre-estrogen receptor (Cre:ER) fusion protein under the transcriptional regulation of the Cytokeratin 14 (K14) promoter, mutant K-Ras^G12D^ expression was induced in murine skin, which resulted in highly penetrant and rapid development of benign hyperplastic oral papillomas. Using a conformation sensitive monoclonal antibody specific for the GTP-bound form of Rac1, we found that these growths had high levels of active Rac1. Genetically removing one Rac1 allele significantly impaired K-Ras induced oral papilloma growth and promoted survival while reducing levels of active Rac1 within the papillomas. These data demonstrate that there is a general dependence of K-Ras on Rac1 to drive proliferation in epithelial tissues *in vivo* and reveal that this requirement is a reflection of Rac activation by K-Ras. Given that inhibitors are being developed to target Rac effector proteins such as PAK kinases [18], [19] or to block Rac activation [20], these results provide compelling target validation of Rac1 in Ras-driven cancers.

## Results

### Mice carrying a LSL-K-Ras^G12D^ allele on a K14-Cre:ER background develop hyperplastic oral papillomas

**Figure 1:**
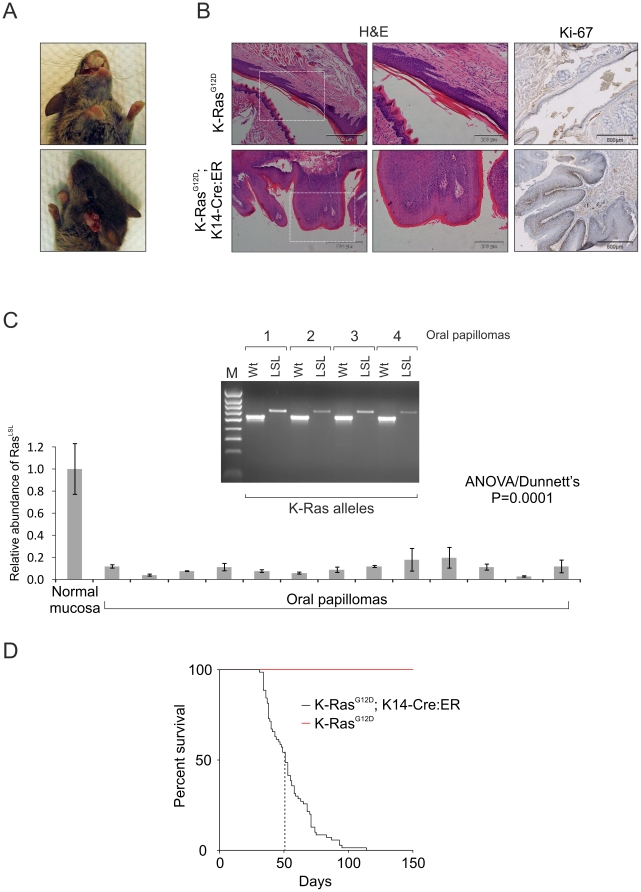
Spontaneous development of papillomas in mice carrying a *LSL-K-Ras^G12D^* allele on a *K14-Cre:ER* background. (**A**) Within weeks of birth *LSL-K-Ras^G12D^;K14-Cre:ER* mice developed papillomas, predominantly in the oral epithelium (upper panel, 78 of 78 mice observed) and occasionally at additional sites such as the side of the head (lower panel, see Table 1 for incidence). (**B**) Hematoxylin and eosin staining (H&E) of oral epithelium shows typical staining in *LSL-K-Ras^G12D^* mice (upper panel) and large intensely staining growths in *LSL-K-Ras^G12D^;K14-Cre:ER* mice (lower panels). Staining for the proliferation marker Ki-67 revealed low levels in *LSL-K-Ras^G12D^* but elevated levels in the proximal regions of papillomas from *LSL-K-Ras^G12D^;K14-Cre* mice. (**C**) Genotyping of genomic DNA from isolated oral papillomas using a wildtype-allele specific primer pair (Wt) and floxed transcription termination cassette-specific primer pair (LSL) showed a reduction in the abundance of LSL amplicon within four independent papillomas (upper panel). This was confirmed by qPCR using the same primer pairs (lower panel). (**D**) Kaplan-Meier survival curve of *LSL-K-Ras^G12D^* and *LSL-K-Ras^G12D^;K14-Cre:ER* mice shows the dramatic differences between each mouse strain. Median survival for *LSL-K-Ras^G12D^;K14-Cre:ER* mice was determined to be 51 days.

In order to generate a model of Ras-driven skin tumorigenesis, we crossed *LSL-K-Ras^G12D^* mice onto a *K14-Cre:ER* background. We anticipated that localized activation of the Cre:ER recombinase by topical application of 4 hydoxytamoxifen (4HT) would result in the genetic recombination event that would remove the transcriptional stop sequence, resulting in expression of the K-Ras mutant G12D allele and ultimately leading to Ras-driven papillomas that would progress to form epidermal carcinomas. However within two weeks of birth, mice heterozygous for the *LSL-K-Ras^G12D^* allele and containing the *K14-Cre:ER* transgene spontaneously developed oral papillomas in the absence of 4HT, suggesting that the Cre:ER fusion protein was not strictly repressed in the absence of ligand (Figure 1A upper panel). On rare occasions, growths were manifested at additional sites such as the side of the head (Figure 1A lower panel and Table 1). Histological analysis of the papillomas revealed that they were well differentiated, papillary and hyperplastic, and Ki-67 staining indicated that they developed by hyperproliferation of the epidermis (Figure 1B). No evidence of invasion or metastases was observed at necropsy or by histology. Every papilloma analyzed (13/13) by a PCR specific for the intact LSL cassette showed spontaneous loss of the LSL cassette that had been knocked into the K-Ras locus [16], consistent with Cre-mediated recombination and expression of K-Ras^G12D^ (Figure 1C). Residual LSL amplicon observed within papillomas is likely the result of stromal cells or inflammatory infiltrate, which are unlikely to exhibit K14-driven Cre-mediated recombination. The large oral papillomas interfered with feeding and *LSL-K-Ras^G12D^*;*K14-Cre:ER* mice did not thrive (Figure 1D). Mice developing oral papillomas were sacrificed when their bodyweight dropped to 80% of that of unaffected littermates. Compared to *LSL-K-Ras^G12D^* mice, which survive well into adulthood, *LSL-K-Ras^G12D^*;K14-Cre:ER mice exhibited an abbreviated lifespan, with a median age of just 51 days (Figure 1D).

**Table 1:** Incidence of oral and other papillomas in *K-Ras^G12D^*;*K14-Cre:ER* mice.

Incidence of Oral papilloma	Other papilloma manifestations		
	Side of head	Urinary	Anal
78/78	4/78	9/78	7/78

### K-Ras driven tumorigenesis is dependent on Active Rac1

**Figure 2:**
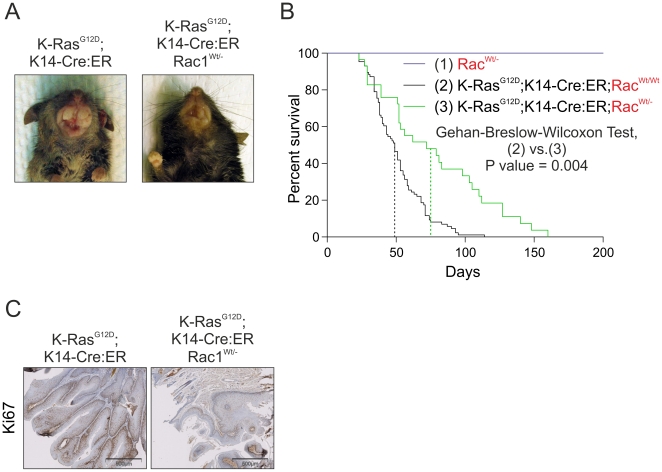
Increased survival of *LSL-K-Ras^G12D^;K14-Cre:ER* mice lacking a Rac1 allele. (**A**) Macroscopic view of oral papillomas from *LSL-K-Ras^G12D^;K14-Cre:ER* (upper panel) and *LSL-K-Ras^G12D^;K14-Cre:ER;Rac1^Wt/-^* mice (lower panel). Deficiency for one Rac1 allele dramatically reduced papilloma growth. (**B**) Kaplan-Meier survival curve of Rac^Wt/-^ (n = 20), *LSL-K-Ras^G12D^;K14-Cre:ER* (n = 78) and *LSL-K-Ras^G12D^;K14-Cre:ER;Rac1^Wt/-^* (n = 22) mice shows the dramatic difference between the two mouse strains. Median survival for *LSL-K-Ras^G12D^;K14-Cre:ER;Rac1^Wt/-^* mice was determined to be 75 days, which is 50% greater than the 51 day survival time of *LSL-K-Ras^G12D^;K14-Cre:ER* mice. (**C**) Staining for the marker of cell proliferation Ki-67 revealed markedly lower levels in tumors from *LSL-K-Ras^G12D^;K14-Cre:ER;Rac1^Wt/-^* mice relative to tumors from *LSL-K-Ras^G12D^;K14-Cre:ER^+^* mice.

It was reported previously that Rac1 was required for K-Ras^G12D^-induced lung cancer [17]. Therefore, we sought to determine whether there was a more general requirement for Rac activity in K-Ras mediated epithelial tumorigenesis. *LSL-K-Ras^G12D^*;*K14-Cre:ER* mice were crossed onto a background lacking one allele of Rac1 [21], which allows for normal birth, development and survival for the duration of the experiment, with no evident abnormalities observed at necropsy (Figure 2B and data not shown). On the Rac1^Wt/-^ background, *LSL-K-Ras^G12D^*;*K14-Cre:ER* mice exhibited dramatically smaller oral papilloma growths and an extended median lifespan of 75 days, representing a ∼50% increase in survival, indicative of slower growing oral papillomas (Figure 2B). Consistent with reduced proliferation being a factor in the *LSL-K-Ras^G12D^*;*K14-Cre:ER;* Rac1^Wt/-^ mice, Ki-67 staining in tumours was markedly reduced compared to those observed from *LSL-K-Ras^G12D^*;*K14-Cre:ER* mice (Figure 2C). These observations indicate that Rac1 contributes to K-Ras mediated epidermal papillomagenesis.

### Rac1 activation by oncogenic K-Ras in vivo

**Figure 3:**
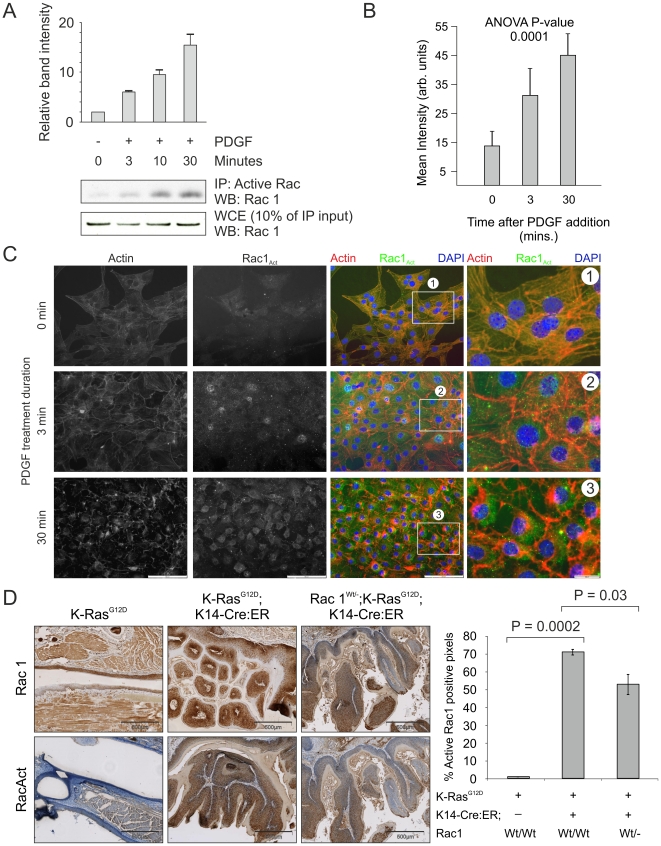
K-Ras activation of Rac *in vivo*. (**A**) Active Rac1 was immunoprecipitated from lysates derived from NIH 3T3 cells that had been treated for the indicated time periods with PDGF (10 ng/ml). Western analysis for Rac1 was carried out on immunoprecipitated protein as well as the original lysate as indicated. (**B**) Histogram showing active Rac1 immunofluorescence intensity averaged over 5 fields of NIH 3T3 cells increasing over the indicated time periods of PDGF treatment. (**C**) Staining for actin with Texas-red conjugated phalloidin (red) and co-immunofluorescence for active Rac1 (green) in representative fields of NIH 3T3 cells used in the quantification shown in panel B, demonstrating increased active Rac1 and changes to the actin cytoskeleton over the indicated time periods of PDGF treatment. Boxed areas indicated by numbers 1-3 are shown enlarged. (**D**) Immunohistochemical analysis for Rac1 and active Rac1 of oral mucosa from LSL-K-Ras^G12D^, LSL-K-Ras^G12D^; K14-Cre:ER and Rac1^Wt/-^; LSL-K-Ras^G12D^; K14-Cre:ER showing dramatic increase in active Rac1 in LSL-K-Ras^G12D^; K14-Cre:ER tissue, which is reduced when one Rac1 allele is lacking. Histogram shows results of positive pixel analysis for active Rac1staining.

Although the requirement for Rac1 in Ras-induced oncogenic transformation has been previously reported [12], [17], there has been no prior demonstration that mutant active Ras increases Rac1 activity in tumors. To this end, we characterized a new commercially available antibody reported to be specific for the active GTP-bound state of Rac1. Using this antibody, we were able to immunoprecipitate active Rac1 in a quantitative way from NIH 3T3 cells that had been treated with PDGF over varying time periods (Figure 3A). In the same lysates, total Rac1 levels remained relatively constant (Figure 3A). We also observed that the immunofluorescence signal from this antibody increased with the duration of PDGF treatment in NIH 3T3 cells (Intensity quantification over 5 fields at Figure 3B and representative fields at Figure 3C). We therefore concluded that this antibody would be useful in determining whether Rac activation occurred in K-Ras-induced tumors. Immunohistochemical analysis of tumors from *LSL-K-Ras^G12D^*;*K14-Cre:ER* mice showed levels of total Rac1 comparable to those observed in healthy normal oral epithelium (Figure 3D). In contrast, the level of active GTP-bound Rac was dramatically increased in K-Ras tumors relative to that in normal oral epithelium (Figure 3D). In all cases of *LSL-K-Ras^G12D^*;*K14-Cre:ER* tumors (>50), we observed a correlative activation of Rac1. Staining was reduced in sections from comparably sized tumors derived from *LSL-K-Ras^G12D^*;*K14-Cre:ER;Rac1^Wt/-^* mice (Figure 3D), indicating that active Rac1 levels were reduced by deletion of one Rac1 allele. Therefore, Rac1 activation is a consistent and highly-reproducible feature of K-Ras activation induced tumorigenesis.

## Discussion

The original intent of this study was to create a genetically modified mouse model in with mutant oncogenic K-Ras^G12D^ would be conditionally expressed following the activation of Cre:ER protein by topical 4HT administration. However, it appears that activity of the gene product from the *K14-Cre:ER* transgene was insufficiently repressed in the absence of ligand, resulting in spontaneous deletion of the LSL cassette and subsequent papilloma development. The effects were likely the consequence of relatively rare events, with the potency of K-Ras^G12D^ in driving keratinocyte hyperproliferation making each occurrence abundantly evident.

The link between Ras and Rac was first proposed following observations that microinjection of recombinant Ras protein produced membrane ruffles which could be blocked by a form of Rac1 deficient in GTP binding said to act as a “dominant-negative” inhibitor [22], [23]. The requirement of Rac1 activity for Ras-mediated oncogenesis was initially reported in 1995 [12], [13], using transformation of NIH 3T3 mouse fibroblasts as the model system and dominant-negative Rac1 as the inhibitor. Further support for Ras activation of Rac came from pull-down assays in which the relatively greater affinity of effector protein Rac-binding domains (RBD) for GTP-bound over GDP-bound was used to selectively enrich for active Rac1 from cell lysates [24], [25]_._

Despite the level of interest in the association of Rac activity with mutant Ras in cancer, there is no data showing that Ras-driven tumors from either mouse or clinical studies are associated with elevated Rac1 activity. One reason is that the methods used to demonstrate Ras-induced Rac activation *in vitro* (e.g. membrane ruffling, pull-downs) cannot be directly converted to use with FFPE tumor samples. A pull-down reagent comprised of the Pak1-RBD was used as an affinity probe in immunohistochemistry experiments to demonstrate Rac activation in FFPE fixed mouse mammary tumors driven by Neu and TGFβ1 [26]. However, given that there have been few attempts to replicate this method (e.g. reference [27]), it does not appear to be easily duplicated. As a result, it would be highly advantageous if a method were developed to detect active Rac1 in tumors, which could be readily deployed using standard immunohistochemistry techniques. We found that an activation-state sensitive Rac1-specific monoclonal antibody performed as a versatile reagent that could immunoprecipitate active Rac1 and detect active Rac1 by immunofluorescence in PDGF-stimulated NIH 3T3 fibroblasts. More importantly, in FFPE sections this antibody was able to detect increased active Rac1 in K-Ras driven oral papillomas relative to normal tissue, which was reduced by the loss of one Rac1 allele. Interestingly, loss of one Rac1 allele did not reduce Rac1 activation within papillomas by 50%, consistent with the requirement for a threshold level of Rac1 activation for papillomagenesis. Our observation that the reduction in Rac1 activation was relatively subtle in Rac^Wt/-^ tumors compared to Rac^Wt/Wt^ tumors, may reflect a positive selection for cells with the greatest levels of activated Rac. In addition, the reduced tumor growth may, at least in part, be a consequence of a lower proportion of cells in the Rac^Wt/-^ tumors being permissive for K-Ras driven hyperproliferation, resulting in an apparent lag that delayed tumor growth. The findings presented in this study could be used to help define the tumor types and potential patient population that would benefit from therapies that target Rac activation or downstream effector signaling pathways.

## Materials and Methods

### Mice

*LSL-K-Ras^G12D^* mice were a gift from David Tuveson (Cambridge Research Institute). Mice carrying the Rac1 null allele and K14-Cre:ER were from Laura Machesky and Owen Sansom. All procedures were performed under appropriate licenses and according to the UK Home Office guidelines.

### Histological and immunohistochemical procedures

Antibodies used were against active Rac1-GTP (Neweast Biosciences cat. no.: 26903), Rac1 (Santa Cruz Biotechnology Inc. cat. no.: sc-217). In both cases, antigen retrieval was carried out by heating sections of FFPE tissue at 99°C for 20 minutes in 10mM citrate buffer, pH 6.0 followed by blocking in 3% hydrogen peroxide in phosphate buffered saline (pH 7.0) for 20 minutes and 5% goat serum/PBS for 20 minutes. Incubation with the appropriate primary antibody was carried out at 4°C overnight in a humidified atmosphere. Following thorough washing in tap water, Envision Dual-link-HRP reagent (Dako) was applied and incubated at room temperature for 1 hour in a humidified atmosphere. After several rounds of washing in tap water, color development was carried out using DAB chromogen (Dako). Slides were counterstained in haematoxylin, dehydrated, cleared and mounted in DPX (Sigma). Slides were imaged using a Hamamatsu Nanozoomer NDP slide scanner (Hamamatsu Photonics) and Digital Slide Server (Slidepath) software.

### Immunofluorescence procedures

Cells were fixed in 4% PFA, permeabilised with 0.5% Triton X-100, and stained in PBS with active Rac1-GTP (Neweast Biosciences cat. no.: 26903) at 1∶500 dilution followed by Alexa 488 fluorophore-conjugated donkey anti–goat antibody (Invitrogen) at 1∶500 dilution. Filamentous actin structures were stained with 1∶250 dilution of Texas Red–conjugated phalloidin (Molecular Probes). Cover-slips were mounted in Vectashield mounting medium containing DAPI (Vector Laboratories).

### Statistical analysis

Unless indicated, statistical significance was determined by Student's t-test. ANOVA was carried out where indicated. In both cases, P<0.05 was used as the cut-off for significance. Data are shown as means ± SD unless otherwise indicated.
